# Some Diseases of the Male Genital System

**Published:** 1908-01-11

**Authors:** 


### SOME DISEASES OF THE MALE GENITAL SYSTEM.

### IV.—THE RARER FORMS OF HYDROCELE.

Congenital hydrocele is a condition in which there is free fluid in the tunica vaginalis, whose cavity communicates with that of the peritoneum. Its name implies that it has existed from birth, and that it is never due to opening up of an unobliterated vaginal process by the pressure of fluid which has accumulated in the tunica vaginalis. Such an explanation is put out of court by the fact that there is nearly always complete obliteration of the process at or near the external abdominal ring. The condition is commonly attributed to a congenital defect in the closure of the vaginal process. In some cases, at any rate, this is the direct result of an undescended testis. Much discussion has arisen as to whether the free fluid originates primarily in the tunica vaginalis or whether it is peritoneal fluid which gravitates into the tunica through the patent opening. There is much to be said for each view; but perhaps the strongest argument in favour of the former is the fact that although the opening is patent from birth, the presence of free fluid may not be noticed until some years after birth, and, when it does make its appearance, it is not associated with ascites or other disease of the peritoneum. Although, as has been said, congenital hydrocele may not make its presence felt for some years, it is usually noticed soon after birth. The ordinary signs of hydrocele, described in a previous article, will be found, and it is not always easy to differentiate between a congenital hydrocele and an ordinary hydrocele of the tunica vaginalis. The points on which reliance must be placed are (1) the more distinct narrowing of the upper pole of the swelling, (2) the communication of an impulse to the hand when the patient coughs (this only occurs if the opening into the peritoneum is moderately wide), and (3) the fact that the fluid can be reduced into the peritoneal cavity by steady pressure. Here again this can only be done if the communication is free. The difficulty of diagnosing the condition from a congenital hernia is even greater. In both there is a scrotal swelling, which may give an impulse on coughing, and may be translucent. But a hydrocele has a smooth regular outline, and goes back very gradually, whereas a hernia gives a doughy sensation to the touch, and goes back with a gurgle. Fortunately the differential diagnosis is nowadays a matter of far less importance than it used to be, since it is now recognised that these scrotal conditions are best treated by open operation; and in any case, now that asepsis can be more or less confidently relied upon, an exploratory incision is a most satisfactory procedure. The treatment of this condition is a simple matter. Some cases in young children do remarkably well with acupuncture and truss pressure, a speedy and permanent cure resulting. Should this fail, there should be no hesitation in advising a radical operation. In no case should a congenital hydrocele in a child be treated by the injection of strong irritants. Sloughing of the scrotum has been known to occur as the result of this form of treatment, and, if the communication with the peritoneum is free, a fatal peritonitis may result. To perform a radical cure an incision should be made over the external abdominal ring as for inguinal hernia. The upper end of the sac of the hydrocele must be carefully isolated from the constituents of the cord and ligatured. The parietal layer of the tunica vaginalis must then be cut away from the testis. This mode of treatment has the great advantage that if a congenital hernia coexists, as it often does, it can be dealt with at the same time. All that it is necessary to do is to open the sac of the hernia, return the intestine into the peritoneum, and ligature the sac high up. It is not only unnecessary, but it is impossible, to perform a set Bassini's operation in young infants on account of the imperfect development of the inguinal canal at that early age.

Infantile hydrocele is a condition in which the tunica vaginalis as well as the funicular process is distended with fluid. But the fluid does not communicate with the peritoneum, as in the congenital variety, because the funicular process is completely occluded at or near the external abdominal ring. In its clinical aspects it strongly resembles an ordinary vaginal hydrocele, especially one whose upper pole comes to a point and reaches up to the external abdominal ring, thus giving a pyriform shape to the tumour. It is recognised by its appearance at a very early age. From the congenital variety it is differentiated by the fact that steady pressure has no influence in reducing its size. The points of differential diagnosis between an infantile hydrocele and an inguinal hernia are the same as those for an ordinary vaginal hydrocele. But once more stress must be laid on the fact that an inguinal hernia in a young child may be quite translucent if the coils of intestine happen to be full of flatus at that moment. The treatment is a simple matter. A great number of these swellings disappear entirely under the influence of evaporating lotion. If this fails acupuncture is almost certain to effect a cure. This slight operation is performed by holding the swelling tense and puncturing it in half a dozen places with an ordinary surgical needle. The fluid escapes partly into the dressings and partly into the cellular tissue, and, curiously enough, does not show any tendency to reaccumulate. More radical treatment is rarely, if ever, called for.

A very interesting variety of hydrocele, but one which is less common in this country than in tropical regions, is the fatty or chylous hydrocele. In this form the tunica vaginalis is filled with a milky-looking fluid, which can be shown microscopically to contain fat globules. Many hypotheses have been advanced to account for this phenomenon. In the tropics it is supposed to be filarial in origin, but it is obvious that this explanation cannot cover cases occurring in northern countries. An alternative theory supposes the chylous fluid to be the result of fatty degeneration of the epithelial cells lining the sac. The treatment does not differ from that of ordinary hydrocele.

